# Clinical features, treatments and prognosis of appendiceal bleeding: a case series study

**DOI:** 10.1186/s12876-023-03025-6

**Published:** 2023-11-03

**Authors:** Xiao-cun Xing, Jin-lin Yang, Xue Xiao

**Affiliations:** 1 Department of Gastroenterology and Hepatology, West China Hospital, Sichuan University, 37 Guoxue Lane, Chengdu, 610041 Sichuan China; 2 Sichuan University–Oxford University Huaxi Gastrointestinal Cancer, Chengdu, China

**Keywords:** Appendiceal bleeding, Obscure gastrointestinal bleeding, Colonoscopy

## Abstract

**Background:**

Appendiceal bleeding is a rare cause of lower gastrointestinal bleeding, could be overlooked and diagnosed as obscure gastrointestinal bleeding. Due to limited real-world cases, the optimized management of appendiceal bleeding is unclear. We here shared our experiences in the past 20 years.

**Methods:**

A retrospective study was conducted at West China Hospital of Sichuan University. We reviewed data of 28,175 colonoscopies from 43,095 gastrointestinal bleeding patients between June 2003 and June 2023. Six patients diagnosed as appendiceal bleeding were included. Data including symptoms, laboratory tests, imaging results, endoscopic findings, treatment and prognosis were collected and analyzed.

**Results:**

Appendiceal bleeding accounts for 0.014% in gastrointestinal bleeding patients. Of the six patients, five were male, with a mean age of 48.5 years. Hematochezia was the most common symptom. The etiology included appendiceal angiodysplasia, appendicitis and appendectomy associated bleeding. Hemostasis was achieved by appendectomy, endoscopic therapy or medication according to different cases. One patient did not receive any treatment because of self-limiting bleeding.

**Conclusions:**

The diagnosis of appendiceal bleeding is challenging, repeated flushing during endoscopy is helpful. Appendectomy is the priority option for treatment as well as the etiology clarification, therapeutic endoscopy and medication could be considered case by case.

**Supplementary Information:**

The online version contains supplementary material available at 10.1186/s12876-023-03025-6.

## Background

Appendiceal bleeding is a rare cause of gastrointestinal bleeding. Due to its anatomical particularity, appendix is hard to be screened under colonoscopy, and its orifice is easily to be overlooked. The misdiagnosis of appendiceal bleeding would result in unnecessary screening of small bowel, as bloody fluid might be found in terminal ileum during colonoscopy. Some of the patients might be diagnosed as obscure gastrointestinal bleeding (OGIB) before appendiceal bleeding was confirmed. Because of the rarity, the treatment preference and prognosis of appendiceal bleeding are unknown. We therefore presented this case series to share the experiences in diagnosing and managing appendiceal bleeding.

## Methods

This study was retrospectively conducted at West China Hospital of Sichuan University, China. We reviewed data of patients who underwent colonoscopy because of gastrointestinal bleeding (GIB) between June 2003 and June 2023. The inclusion criteria were adult patients who had hematochezia or melena, and appendiceal bleeding was diagnosed by colonoscopy. Patients were excluded if the bleeding resulted from multiple lesions besides appendix. All the inpatient and outpatient data were recorded in the Hospital Information System and collected. Variables including demographics, symptoms, laboratory tests, imaging results, endoscopic findings, treatment and prognosis were analyzed. Olympus 260 and 290 series, including esophagogastroduodenoscopy (OGD), colonoscope and single balloon assisted enteroscope (Olympus, Tokyo, Japan) were used. All of the endoscopists have performed more than 1,000 OGDs and 500 colonoscopies. Clinical success was defined as no recurrent appendiceal bleeding in the following 30 days after treatment. Continuous variables were expressed as mean ± standard deviation (SD) and categorical variables were presented as absolute values and percentages. All statistical analyses were performed using IBM SPSS software, version 26.0 (IBM Corp., Chicago, IL).

This study was approved by Medical Ethics Committee of West China Hospital, and informed consents were obtained from the patients for the publication of their information and imaging.

## Results

In the past 20 years, 43,095 gastrointestinal bleeding patients received endoscopy examination (including OGD, colonoscopy, sigmoidoscopy, balloon-assisted-enteroscopy and capsule endoscopy) at West China Hospital of Sichuan University. In this study, we retrospectively reviewed data of 28,175 colonoscopies performed in the above patients, and 6 patients were identified as appendiceal bleeding, accounting for 0.014% of bleeding reasons.

### Appendiceal angiodysplasia

#### Case 1

**Fig. 1:**
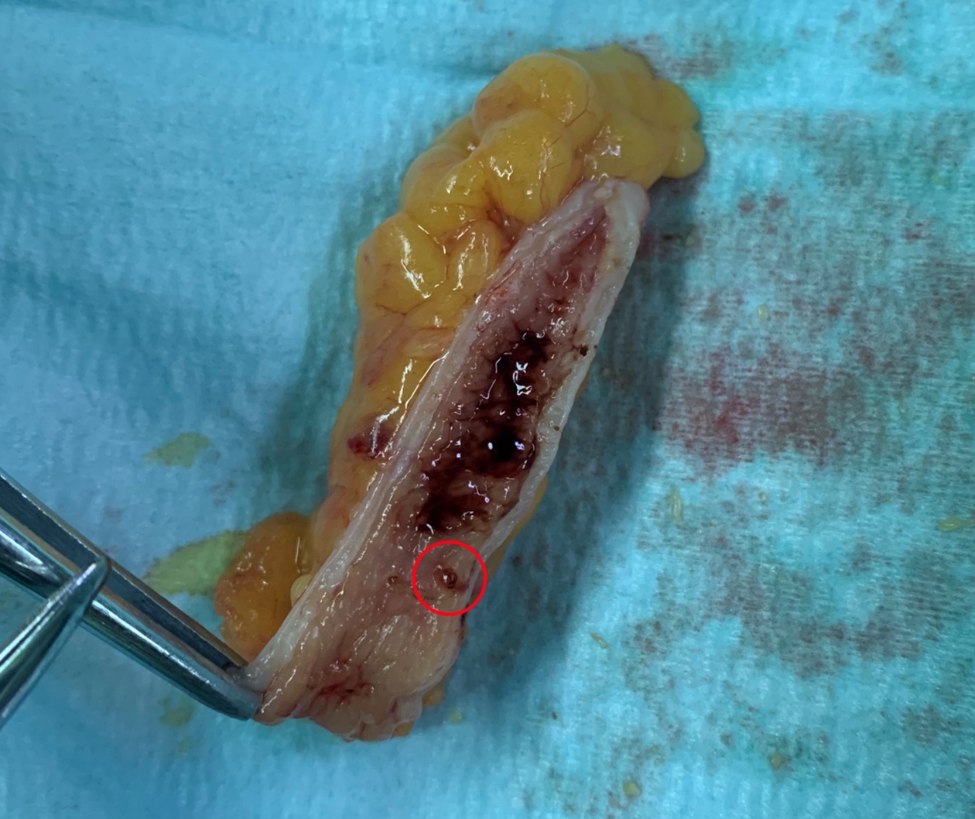
Appendiceal angiodysplasia Case 1: After cutting and dissected, malformation vessel was found in the mucosa of appendix (red circle)

A 63-year-old man was admitted because of periodic hematochezia for 30 years, which occurred every 3–4 years after spicy food taken and recurred 6 days ago. Repeated OGD, colonoscopy and capsule endoscopy did identify any lesions. Therefore, he was diagnosed as OGIB. On admission, his vital sign was stable with hemoglobin (HB) concentration of 105 g/L. He denied any other diseases or operation history. During hospitalization, medication treatment failed to achieve hemostasis, and HB concentration decreased to 81 g/L. Colonoscopy showed active bleeding at the appendix orifice after repeated water flushing and screening (Additional Video 1). The patient received appendectomy. There was no appearance of appendicitis. After dissecting the appendix, blood clots were noted in the lumen, and a malformed vessel was found (Fig. 1). The postoperative pathology rejected any other pathological changes. The patient was diagnosed as appendiceal angiodysplasia. No recurrent bleeding occurs in the following 2 years.

#### Case 2

**Fig. 2:**
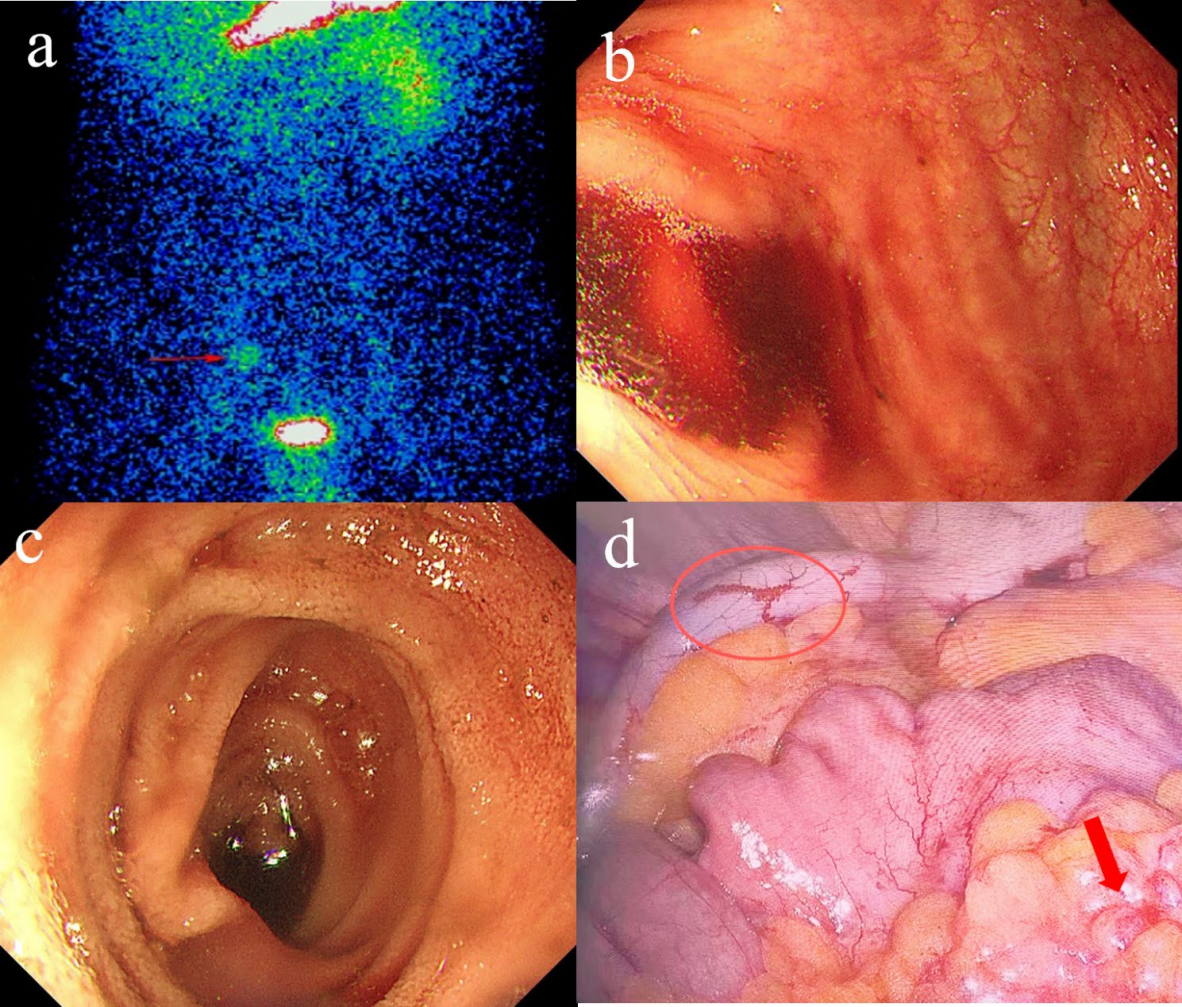
Appendiceal angiodysplasia Case 2: (**a**) Abnormal radioactive concentration in the ileocecal region on SPECT imaging of Meckel’s diverticulum. (**b**) A large amount of blood noted throughout the colon lumen. (**c**) No blood found in the proximal ileum. (**d**) During laparoscopy, varices-liked vascular malformation (red circle) was found on the outside surface of the appendix, and a diverticulum (red arrow) was found at the distal ileum

A 33-year-old man was admitted because of recurrent bloody stools for more than two years. His stool could turn into yellow after medication therapy. OGD, colonoscopy, balloon-assisted enteroscopy and capsule endoscopy were failed to identify the bleeding lesion. He was diagnosed as OGIB. On admission, his vital sign was stable with HB concentration of 137 g/L. Single photon emission computed tomography (SPECT) indicated Meckel’s diverticulum (Fig. 2a). During hospitalization, hemocoagulase and somatostatin failed to achieve hemostasis. Retrograde balloon-assisted enteroscopy revealed bloody colon and terminal ileum (Fig. 2b), no blood in the proximal ileum (Fig. 2c), and failed to find the diverticulum. After repeated flushing, fresh blood was found to flow from the appendix orifice (Additional Video 2). In view of uncontrolled bleeding, laparoscopy was performed. During operation, varices-liked vascular malformation was noted on the outside surface of the appendix, also a diverticulum was found at the distal ileum (Fig. 2d). Blood clots were found only in the appendix lumen other than the diverticulum lumen. Histology examination ruled out other pathologies of appendix. In the following 3 months, no rebleeding occurred.

### Appendicitis associated bleeding

**Fig. 3:**
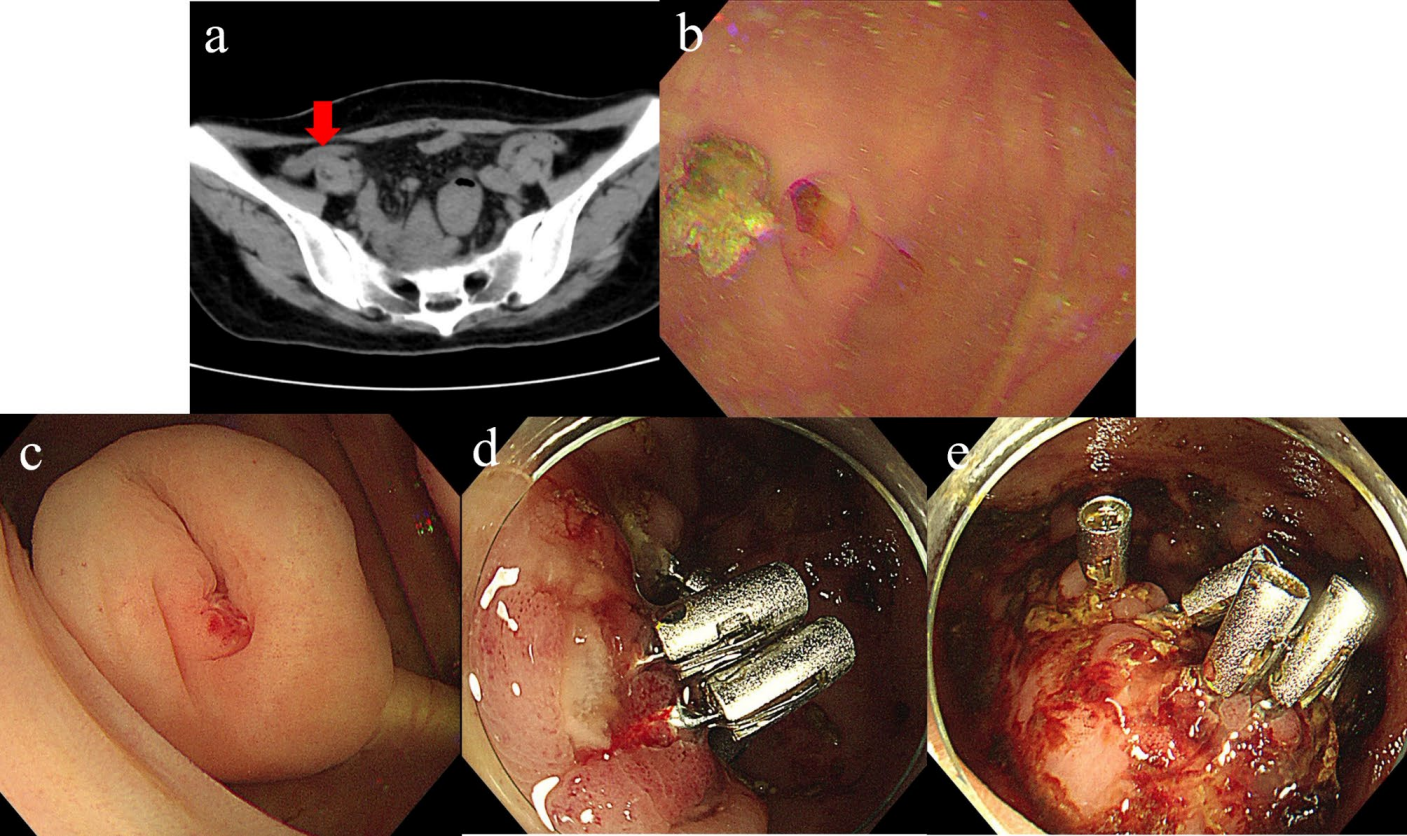
Appendicitis associated bleeding: (**a**) A long thickening appendix (red arrow) was shown in the contrast-enhanced CT image. (**b**) After removing the fecaloma away, fresh blood flowed from the orifice of appendix. (**c**) Ulcer with visible vascular stumps at the edge of the remaining appendix was seen. (**d-e**) Multiple hemostatic clips with 0.2-0.4 cm oozing ulcers were seen at the basement

A 48-year-old woman was admitted because of melena for an hour. She was diagnosed as undifferentiated sarcoma-prone right maxillary tumor and had received surgical operation and postoperative radiotherapy and chemotherapy three months ago. On admission, her vital sign was stable and HB concentration was 58 g/L. Contrast-enhanced computed tomography (CT) showed a long thickening appendix (Fig. 3a). Initial OGD and colonoscopy revealed no bleeding lesions. During retrograde balloon-assisted enteroscopy, intermittent fresh blood was found to flow from appendix orifice (Additional Video 3). After flushing, a fecaloma moved out to the orifice and was removed, then we observed oozing and a 0.5 cm ulcer in appendix lumen (Fig. 3b and c). Medication treatment could achieve hemostasis, but hematochezia recurred after suspending somatostatin. Appendectomy was then performed. Postoperative histology showed acute uncomplicated appendicitis. On the third day after operation, bloody stool recurred. Colonoscopy revealed ulcer with visible vascular stumps at the edge of the remaining appendix (Fig. 3d and e). Hemostasis with electrocoagulation and clips was done, but failed. Angiography showed no abnormality. After consultation, right hemicolectomy was suggested by surgeon. Contrast-enhanced CT was reexamined, and revealed tumor metastasis in abdominal and pelvic cavity. The patient preferred chemotherapy and refused further surgical treatment. Her stool gradually turned into yellow after chemotherapy, and no rebleeding occurred in the next 3 months.

### Post-appendectomy bleeding

**Fig. 4:**
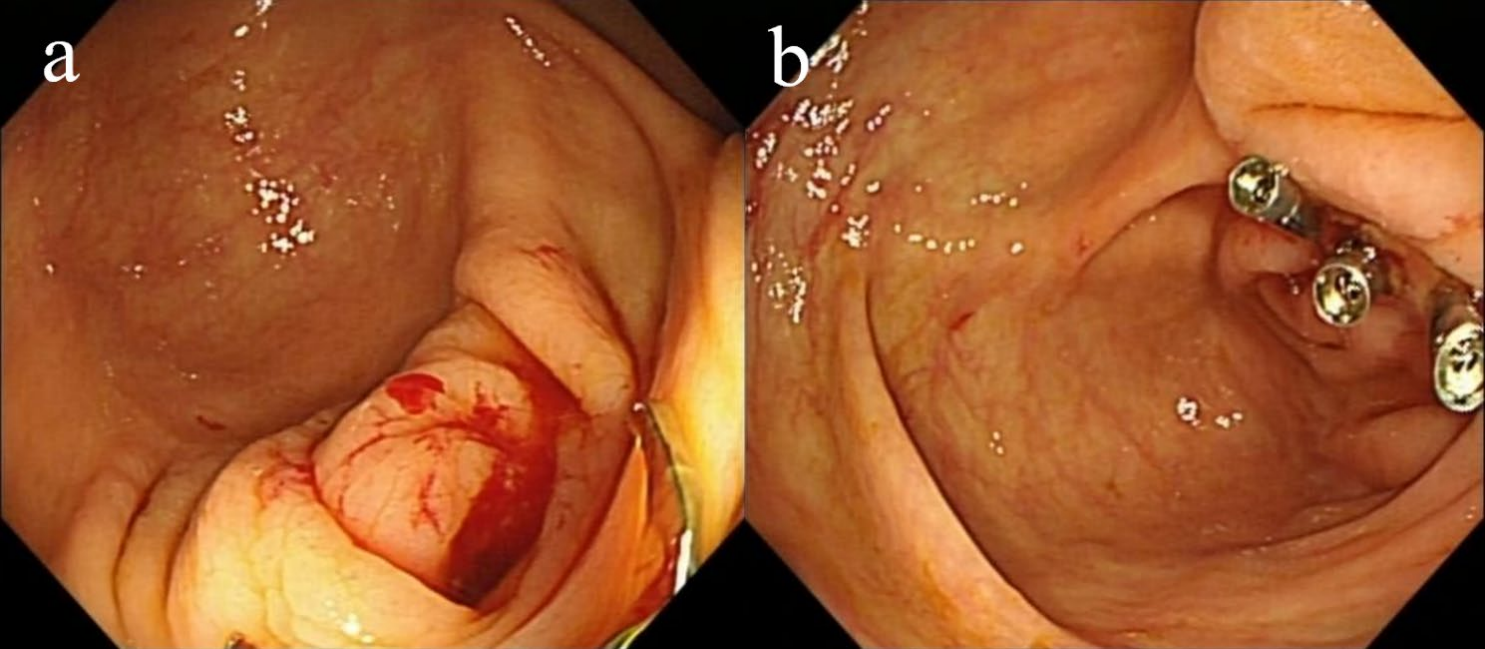
Post-appendectomy bleeding: (**a**) Fresh blood was found at the post-appendectomy wound. (**b**) Three clips were used to close the appendiceal foramen

A 67-year-old man was admitted because of intermittent hematochezia for 13 days, and he had received appendectomy due to acute gangrenous appendicitis 17 days ago. On admission, his vital sign was stable. There was no abdominal tenderness or rebound tenderness, and bowel sounds were brisk. Laboratory test showed a HB concentration of 67 g/L, and the CT images indicated peritoneum thickening, bowel wall and mesenteric swelling. OGD showed no signs of bleeding. Colonoscopy revealed post-appendectomy wound with oozing after water flushing (Fig. 4a). Three clips were used to close the appendix orifice and bleeding stopped (Fig. 4b). He was discharged then. No rebleeding occurred in the 4 years of follow-up.

### Appendiceal bleeding of unknown reason

#### Case 1

**Fig. 5:**
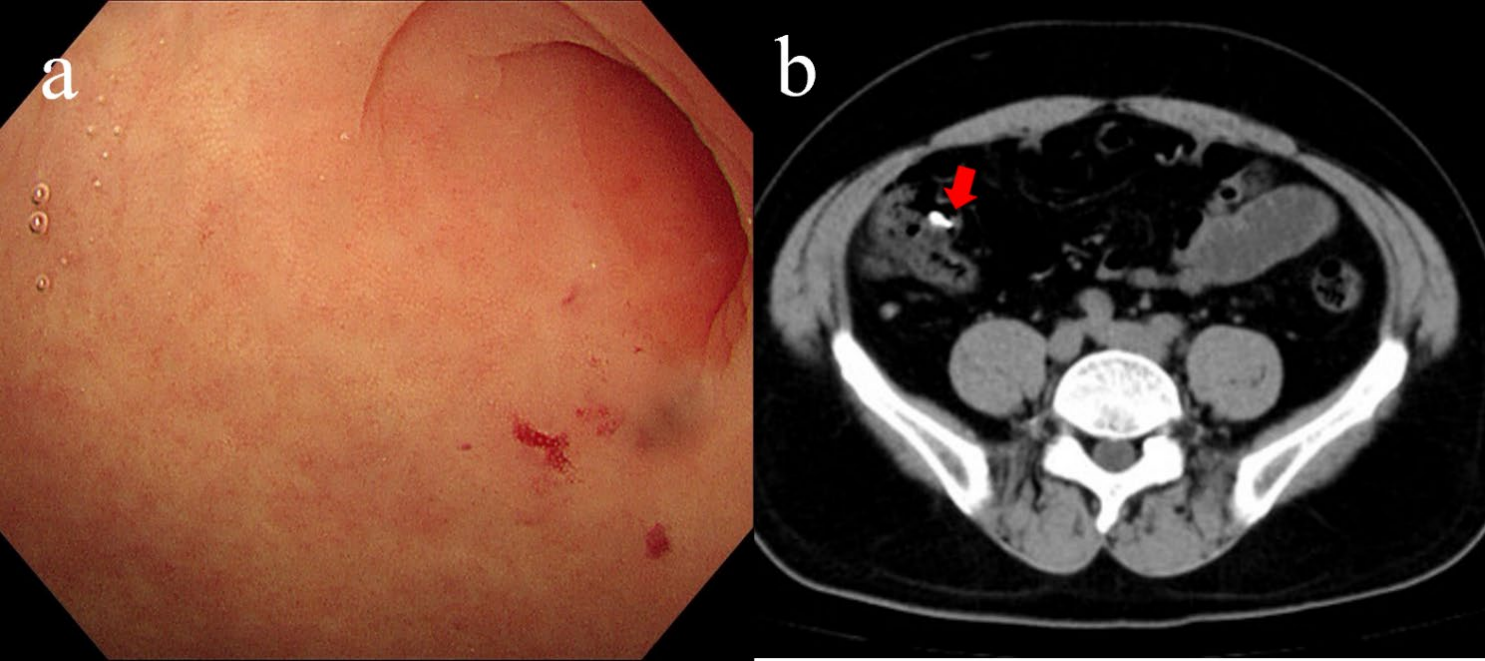
Appendiceal bleeding of unknown reason Case 1: (**a**) Bleeding was found at the appendix orifice after flushing and waiting. (**b**) Fecaloma (red arrow) was showed in contrast-enhanced CT

A 42-year-old man complained of intermittent melena and hematochezia for two days. He had received OGD and colonoscopy at outpatient clinics. OGD revealed no abnormalities, and colonoscopy revealed fresh blood in the cecum. After flushing, intermittent bleeding was found at the appendix orifice, and the surrounding mucosa was normal (Fig. 5a, Additional Video 4). Fecaloma was indicated in contrast-enhanced CT scan (Fig. 5b) and no findings was found in SPECT Meckel’s diverticulum imaging. He had no anemia and denied any other medical history. The patient did not take any treatments. In the follow-up 4 months, there were no recurrent bleeding.

#### Case 2

**Fig. 6:**
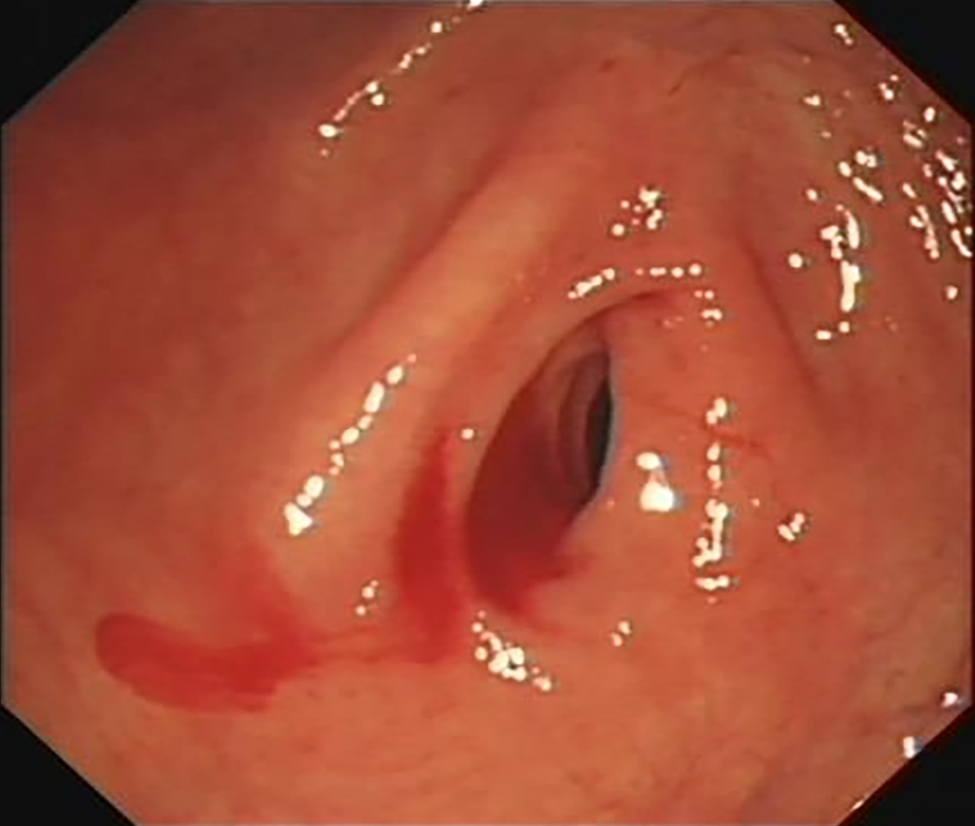
Appendiceal bleeding of unknown reason Case 2: Fresh blood was found at the appendix orifice

A 38-year-old man was admitted due to hematochezia for three days. His HB concentration was 155 g/L and stool occult blood test was positive. Emergent colonoscopy revealed bloody cecum, and fresh blood draining from the appendix orifice (Fig. 6). Contrast-enhanced CT did not show any abnormality. Medication treatment including carbazochrome sodium sulfonate and hemocoagulase was given and bleeding stopped. The patient was discharged 5 days later. No rebleeding occurred over the 5-year follow-up.

### Case summary

The average age of the patients was 48.50 ± 13.75 years, ranged from 33 to 67, and the majority (83.7%) was male. Hematochezia was the most common symptom. Half of the patients developed anemia, and the average of HB is 104.40 ± 42.35 g/L. Five of them had Oakland scores greater than 8, with the average value of 18.17 ± 9.83. The confirmed etiology of appendiceal bleeding included appendiceal angiodysplasia, appendicitis associated bleeding and post-appendectomy bleeding (Table 1). Hemostasis was achieved in two appendiceal angiodysplasia patients by appendectomy, one post-appendectomy bleeding patient by endoscopic therapy, one patient by medication treatment, and one self-limiting bleeding without further treatment. The case of appendicitis associated bleeding with severe comorbidity finally achieved hemostasis after appendectomy, endoscopic hemostasis and medication treatment. Clinical success was achieved in all patients.

**Table 1:** Clinical characteristics of 6 patients of appendiceal bleeding

Patient	Age (Years)	Sex	Symptom	HB concentration (g/L)	Oakland score	Duration of symptoms	Findings of CT scan	Times of endoscopy examination				Etiology	Therapy	Clinical success
								OGD	Colonoscopy	Capsule endoscopy	Balloon-assisted-enteroscopy			
1	63	M	Hematochezia	105	22	30 years	No abnormality in appendix	3	3	1	0	Appendiceal angiodysplasia	Appendectomy	Yes
2	33	M	Hematochezia	137	12	2 years	No abnormality in appendix	2	2	2	2	Appendiceal angiodysplasia	Appendectomy	Yes
3	48	F	Hematochezia &Melena	58	31	1 day	Long thickening appendix	2	4	0	1	Appendicitis associated bleeding	Appendectomy & Endoscopic hemostasis & Medication	Yes
4	42	M	Hematochezia &Melena	135	8	2 days	Fecaloma in appendix	1	1	0	0	Suspected of appendicitis	None	Yes
5	38	M	Hematochezia	155	9	3 days	No abnormality in appendix	1	1	0	0	Unknown	Medication	Yes
6	67	M	Hematochezia	67	27	13 days	No abnormality in appendix	1	1	0	0	Post appendectomy	Endoscopic hemostasis	Yes

HB, blood hemoglobin; OGD, esophagogastroduodenoscopy; M, male; F, female

## Discussion

This report presented a rare case series of appendiceal bleeding, and we found it accounting for 0.014% gastrointestinal bleeding reasons. Its misdiagnosis leads to unnecessary small bowel examination, because bloody fluid could be noted in terminal ileum during colonoscopy and intermittent bleeding at appendix orifice could be overlooked. In this study, half of the patients experienced capsule endoscopy or enteroscopy. Our experience indicated that, if fresh blood was found in terminal ileum, repeated water flushing was helpful to confirm whether appendiceal bleeding exited. Previous case reports indicated contrast-enhanced CT and angiography might be helpful, but positive findings need a bleeding rate of more than 0.5ml per minute, which is a severe bleeding [1–4]. Our study showed that majority patients experienced mild bleeding, and CT scan as well as angiography did not work very well.

The etiology of appendiceal bleeding have been reported to include appendicitis, diverticulum, angiodysplasia, inflammation bowel disease, endometriosis, tumor, tuberculosis and dieulafoy’s disease, in which appendicitis is the most common one [1, 5–10]. Some of appendiceal bleeding cases is acute, but most of the them is chronic and insidious [5]. In our case series, angiodysplasia and appendicitis were the most common reasons.

The incidence of angiodysplasia related lower gastrointestinal bleeding ranges from 3 to 40% [11], and it rarely occurs in appendix. Patient with appendiceal angiodysplasia is hard to be recognized by colonoscopy [8]. In our cases, both patients of appendiceal angiodysplasia were ever misdiagnosed as OGIB, and appendectomy was the effective treatment. In the previous reported angiodysplasia case, transcatheter arterial embolization was tried but failed to achieve hemostasis. We consider the angiodysplasia might not come from artery, therefore arterial embolization would not work [8].

In patients with appendicitis, the hemostasis effect of appendectomy was limited. Part of the appendix was remained after operation, as described in the case of appendicitis associated bleeding, the bleeding lesion was not treated effectively. However, the case of appendicitis associated bleeding had severe comorbidities. Previous study has reported that poor physical condition increases post-appendectomy mortality and the risk of complication [12]. On the other side, appendectomy can also induce postoperative appendiceal bleeding [13]. But after appendectomy, endoscopic hemostasis could be performed without concerns of secondary appendicitis. We do not recommend transcatheter arterial embolization, as it could induce ischemic change, and increase the risk of perforation in appendicitis patients [14].

Medication, especially somatostatin, might show good control in acute bleeding condition, but cautious need to be paid for rebleeding.

Besides, appendiceal bleeding could be self-limiting. Two young patients in our study experienced a short-term hematochezia without other discomforts. The bleeding was mild, and stopped spontaneously or after nonspecific medication treatment. This might partially explain why appendiceal bleeding is rare, and its incidence may be underestimated. Given the young age of the patients, appendicitis associated with mucosal bleeding might be the most likely reason.

## Conclusion

Conclusively, the prognosis of appendiceal bleeding is good, but diagnosis is challenging. Repeated flushing and meticulous observation during colonoscopy might be helpful to confirm the diagnosis. Appendectomy is the primary option treatment as well as clarifying the diagnosis. However, the therapeutic choice should be tailored to individual patient.

### Electronic supplementary material

Below is the link to the electronic supplementary material.

Supplementary Material 1

Supplementary Material 2

Supplementary Material 3

Supplementary Material 4

Supplementary Material 5

## Data Availability

All data generated or analysed during this study are included in this published article and its supplementary information files.

## Abbreviations

OGIB: Obscure gastrointestinal bleeding
GIB: Gastrointestinal bleeding
OGD: Esophagogastroduodenoscopy
HB: Blood hemoglobin
SPECT: Single photon emission computed tomography
CT: Computed tomography
