# Supplementary material for: Clinical features, treatments and prognosis of appendiceal bleeding: a case series study
Source: BMC Gastroenterol. 2023 Nov 3;23:377. doi: 10.1186/s12876-023-03025-6 (PMC10625278; doi:10.1186/s12876-023-03025-6)
Supplement: Supplementary file 5 — Supplementary Material 5 [file 12876_2023_3025_MOESM5_ESM.docx]

**Additional files**:

**Additional Video 1** Appendiceal angiodysplasia Case 1: Active bleeding at the appendix orifice after repeated flushing.

**Additional Video 2** Appendiceal angiodysplasia Case 2: Active bleeding at the appendix orifice after repeated flushing.

**Additional Video 3** Appendicitis associated bleeding: Intermittent fresh blood flowed from appendix and oozing erosion at the appendix orifice noted.

**Additional Video 4** Appendiceal bleeding of unknown reason Case 1: Intermittent bleeding was found at the appendix orifice after repeated flushing.
